# Natural history of gait patterns in untreated children with bilateral cerebral palsy in a low‐income country setting

**DOI:** 10.1111/dmcn.16113

**Published:** 2024-10-11

**Authors:** Julie Stebbins, Laurence Wicks, Tim Nunn, Richard Gardner, Tewodros T. Zerfu, Mesfin Kassahun, Tim Theologis

**Affiliations:** ^1^ Oxford Gait Laboratory Oxford University Hospitals NHS Foundation Trust Oxford UK; ^2^ Nuffield Department of Orthopaedics, Rheumatology and Musculoskeletal Sciences Oxford University Oxford UK; ^3^ Orthopaedic Surgery Department CURE Children's Hospital of Ethiopia Addis Ababa Ethiopia; ^4^ Orthopaedic Surgery Department Hospital for Sick Children Toronto Canada; ^5^ Department of Surgery University of Toronto Canada

## Abstract

**Aim:**

To assess a group of ambulant, untreated children with bilateral spastic cerebral palsy, in a resource‐poor setting, who had never been assessed by a health care professional or received any treatment, to help establish the natural history of gait patterns in this condition.

**Method:**

At CURE Children's Hospital of Ethiopia, 46 children with no prior health care contact were assessed in a cross‐sectional cohort study, through a detailed history, clinical examination, and instrumented gait analysis using a motion capture system.

**Results:**

There was a large spread in the data reflecting the high natural heterogeneity in this population. The severity of gait pathology did not correlate with age; however, a small but significant reduction in sagittal hip and knee range of motion with increasing age was observed. There was also a trend towards reduced passive knee extension with age.

**Interpretation:**

Improved understanding of the aspects of gait that are likely to naturally improve, deteriorate, or remain stable over time helps guide treatment decisions in this population.

**Abbreviation**
GPS: Gait Profile Score

**What this paper adds**

- Large variability was present in gait pattern across different ages.
- Severity of gait pathology and walking speed was not correlated with age.
- There was small but significant reduction in dynamic hip and knee motion with age.
- There was a trend towards reduced passive knee extension with age.

Cerebral palsy (CP) is a broad diagnosis that encompasses a group of disorders affecting movement and posture, caused by pathology within the brain during either the prenatal or the early infancy period. The resulting presentation varies significantly between individuals, and is determined by the timing, site, and extent of the brain lesion.^1^

While the initial brain lesion remains static over time, there are progressive changes to the musculoskeletal system, due to the cascade of altered motor control affecting abnormal muscle activation and subsequently impacting on bone growth throughout childhood. However, the natural history of this condition is poorly understood as longitudinal studies investigating progression in untreated children are limited by ethical considerations. Withholding all musculoskeletal and neurological interventions to assess natural progression cannot be justified. This is because it is widely accepted that, in the absence of intervention, musculoskeletal deformities progress over time.

Given this limitation, much of the evidence regarding the natural history of CP is cross‐sectional in nature, and largely only excludes surgical intervention, while interventions such as orthotic management, casting, physiotherapy, and even sometimes spasticity management, are included within the ‘natural history’ cohort.

The evidence currently available suggests that the Gross Motor Function Measure tends to improve and then plateau around 7 to 8 years of age in less affected children (Gross Motor Function Classification System [GMFCS] levels I and II), while it tends to deteriorate slightly from this age onwards in more severely affected children (GMFCS levels III, IV, and V).^2^ Gait patterns have been reported to deteriorate with time in the absence of orthopaedic surgery, including a progressive reduction in walking speed,^3^, ^4^ as well as increasing knee flexion during the stance phase of gait (crouch pattern).^5^ In addition, reduced range of motion in the sagittal plane at the hip, knee, and ankle, increasing hip adduction, increasing external rotation of the tibia, and internal rotation of the foot^4^ have been reported.

Passive range of lower limb joint motion has also been reported to reduce over time, particularly in terms of increasing popliteal angle, reduced hip abduction,^3^, ^4^ and reduced ankle dorsiflexion.^3^ However, the current evidence in the literature largely depicts the progression of musculoskeletal deformity in the context of non‐surgical/conservative management, rather than true natural history.

Orthopaedic surgery is a common intervention in children with CP. Overall, gait patterns have been shown to improve in both the short and longer term after common interventions.^6^, ^7^, ^8^ However, there is also reported variability in outcomes between individuals with a relatively low level of evidence on which to base practice.^9^ Presumably, some of this variability is due to a lack of information on the natural history of the condition, which in turn limits the predictability of surgical outcomes.^9^ Improved understanding of natural history of gait pathology and its association with the neurological and musculoskeletal aspects of the child's presentation could help with appropriate selection of children for specific interventions and therefore improved outcomes overall.

CP is prevalent in low‐ to middle‐income countries, with current evidence suggesting a possibly even higher rate than in high‐income countries.^10^ Often, children with CP present to health care professionals at a much later age, having had no previous intervention.

The aim of this study, therefore, was to assess the data on a cross‐sectional cohort of children with bilateral spastic CP in a low‐income country (Ethiopia), who are naive to any neurological, musculoskeletal, or any other health care interventions, thus representing a true natural history of the condition, to provide a baseline for future assessment of treatment outcomes.

## METHOD

### Setting

Data were collected between 2019 and 2024 in this cross‐sectional, cohort study, in the gait laboratory at CURE Children's Hospital of Ethiopia. This laboratory was set up in collaboration with the team from the Oxford Gait Laboratory and continues to work in close collaboration. All protocols are in line with Oxford Gait Laboratory, to facilitate ongoing support. In addition, the gait laboratory is undergoing an accreditation process under the Clinical Movement Analysis Society of UK and Ireland.^11^ There are routine assessments of repeatability of marker placement, clinical examination, and data processing as part of this process. Only measures that demonstrated evidence of repeatability within the thresholds specified by the Clinical Movement Analysis Society of UK & Ireland were included in this study to ensure confidence in the data.

### Participants

All children included in this study were seen by CURE Children's Hospital of Ethiopia clinicians—either at the outpatient department in Addis Ababa, Ethiopia, or were identified during outreach clinics around Ethiopia and referred to Addis Ababa for gait analysis and further management. All children that were being considered for surgical management were referred, along with those who had a question related to orthotic management.

Inclusion criteria were a diagnosis of bilateral spastic CP, GMFCS levels I to III, aged between 6 to 18 years, and naive to any medical, neurological, or musculoskeletal interventions (i.e. first presentation to a health care professional). The diagnosis of CP could only be made based on reported history in this study, as brain magnetic resonance imaging is not routinely undertaken in Ethiopia. However, all participants were carefully screened to eliminate any other potential diagnosis, and ensure their history was consistent with this diagnosis.

Exclusion criteria were any concomitant medical condition affecting gait, or the inability to complete instrumented gait analysis.

All parents/carers of the participants signed informed consent, and the study was approved by the CURE Children's Hospital of Ethiopia local ethical board (CNR/05/24).

### Data collection

A detailed clinical history was recorded for all children, including birth history, development of motor milestones, and current functional ability. A standardized physical assessment was performed in 43 of the participants (physical examination was not performed on three participants) to determine passive joint range of motion for the lower limbs, manual muscle testing of strength using the Medical Research Council scale, assessment of torsional profile, including femoral anteversion using the trochanteric prominence angle test,^12^ and qualitative categorization of foot posture based on visual assessment.

Instrumented gait analysis was undertaken at the CURE Children's Hospital of Ethiopia Gait Laboratory in Addis Ababa. Passive reflective markers were applied to anatomical landmarks according to a modified conventional gait model.^13^ Pelvis angles were calculated according to Baker^14^ which calculates the sequence of pelvic rotations in the order of transverse plane, frontal plane, and then sagittal plane. Hip joint centres were calculated according to the regression equation reported by Harrington et al.^15^ as this has been shown to be most accurate when used with children and individuals with CP, and knee angles were calculated using the Symmetrical Axis of Rotation Approach which is a functional method first reported by Ehrig et al.^16^ Kinematic data were recorded using a 10‐camera Bonita system (Vicon, Oxford, UK) recording at 100 Hz, while children walked barefoot at self‐selected speed across a level walkway. They were asked to walk in their typical manner, using whatever walking aids they would normally use. A minimum of six trials were collected to ensure representative data was collected.

The Gait Profile Score (GPS)^17^ was calculated using six representative trials from each child. The GPS is a single number score which depicts the degree of gait abnormality by computing the root mean square error between the patient's data and that of a cohort of age‐ and sex‐matched children with no gait pathology. This cohort of children with no gait pathology included 20 children aged 6 to 18 years (13 males, seven females); their gait data was collected locally at the CURE Children's Hospital of Ethiopia gait laboratory. The reference data included two groups (10 children aged 6–9 years and 10 children aged 10–17 years) to conform to the Clinical Movement Analysis Society of UK & Ireland standards for clinical gait analysis.^11^

### Statistical analysis

The relationship between GPS and age was investigated using Pearson correlation (SPSS version 29; IBM Corp., Armonk, NY, USA), along with walking speed (normalized to leg length and reported as a percentage of normal speed by comparing to age‐ and sex‐matched data). In addition, specific gait variables were assessed to determine their relationship with age, focussing on those previously identified in the literature (maximum knee extension in stance, range of motion in the sagittal plane for the hip and knee, average ankle dorsiflexion, average hip adduction, and average foot progression angle). Partial correlation was used for this assessment, with the individual as a covariate to account for the fact that both legs of each individual were included in the analysis.

Finally, passive joint ranges of motion and femoral anteversion were also assessed to determine their relationship with age using a similar statistical approach to that used for the specific gait variables. Significance was set at a *p*‐value lower than 0.05. To determine the prevalence of different types of foot posture, the cohort was split into three age brackets to allow roughly even numbers in each group (6–10 years, 11–13 years, 12–18 years) and the tally for each foot type (based on visual observation) was recorded for each group.

## RESULTS

In total, 46 children were included in this study (age 6–18 years, 31 males, GMFCS level I = 3, GMFCS level II = 32, GMFCS level III = 11).

**FIGURE 1:**
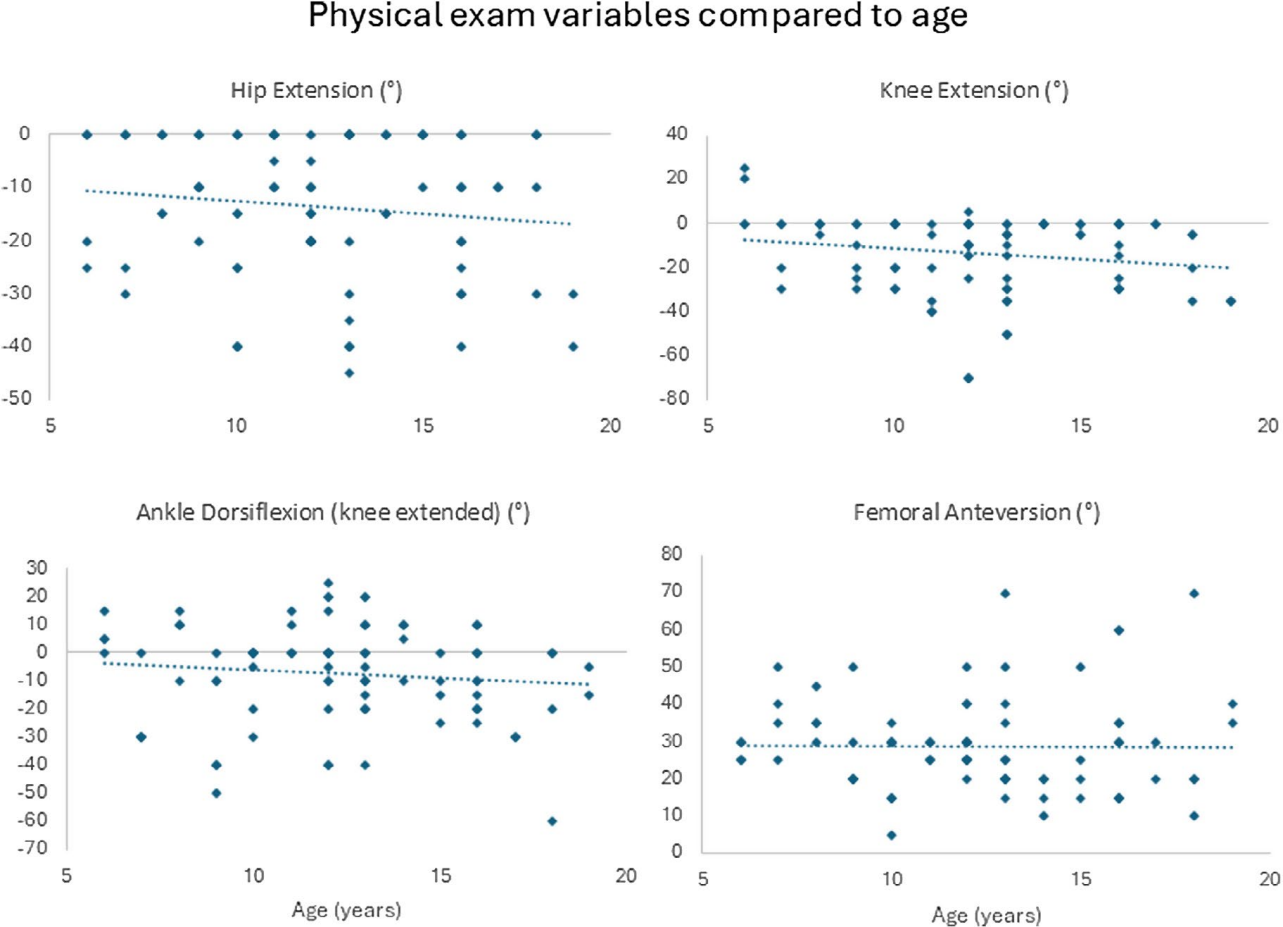
Physical exam variables compared to age. Negative values for hip extension, knee extension, and ankle dorsiflexion indicate joint contracture.

**FIGURE 2:**
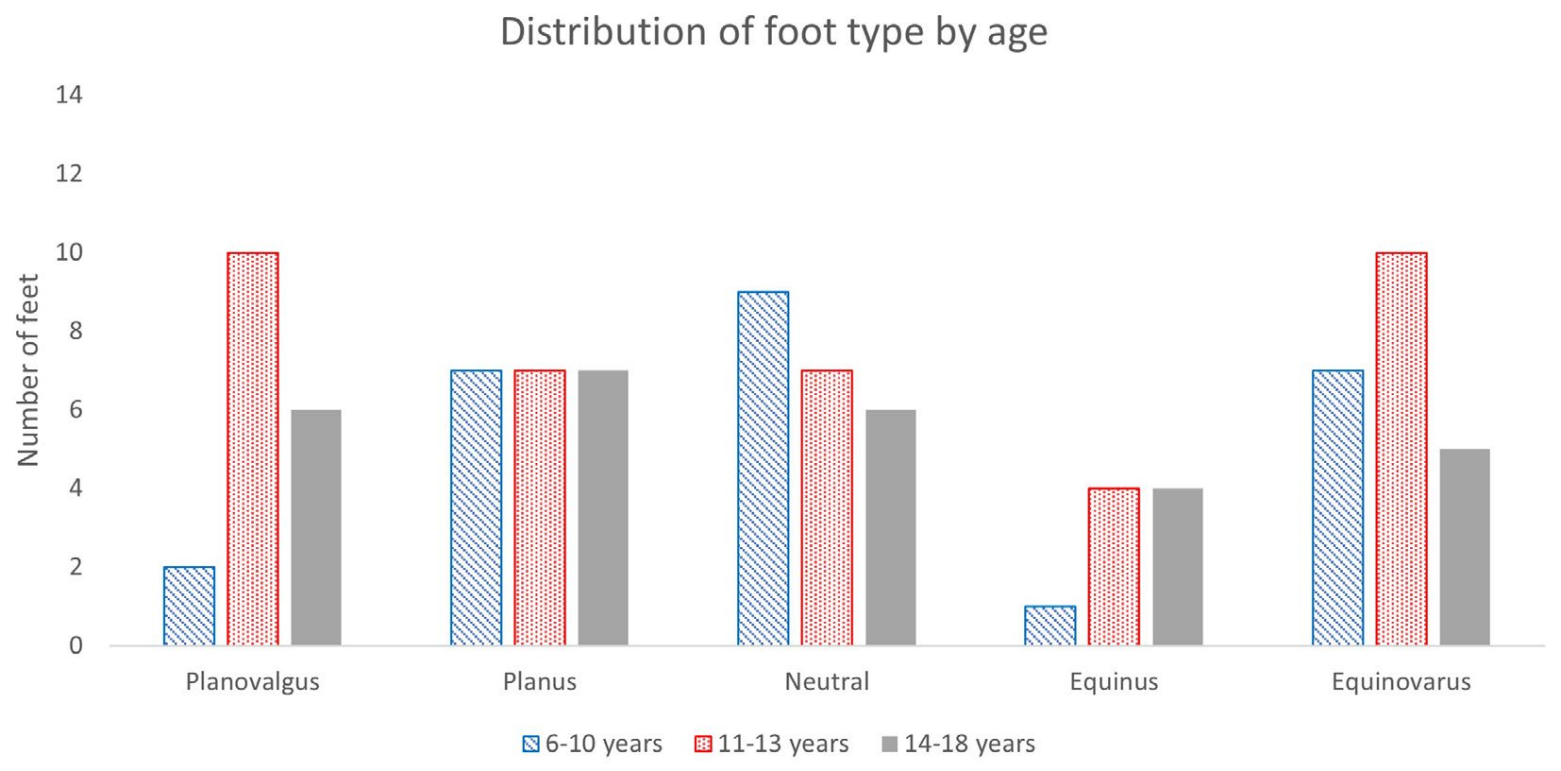
Distribution of different foot types in three different age groups (6–10 years, 11–13 years, and 14–18 years) (*n* = 92 feet).

On clinical examination, only joint ranges of motion were retained for further analysis, along with femoral anteversion, because of reliability issues with the other measures. The results from the physical assessment showed that none of the reported measures significantly correlated with age (hip extension *R* = −0.141, *p* = 0.216; knee extension *R* = −0.187, *p* = 0.094; ankle dorsiflexion R = −0.104; *p* = 0.364; femoral anteversion *R* = −0.020, *p* = 0.364) (Figure 1). There was large inter‐individual variation for all measures (hip extension range: −45° to 0°; knee extension range: −70° to 25°; ankle dorsiflexion: −60° to 25°; femoral anteversion: 5° to 70°). The incidence of different foot types was relatively evenly distributed across the younger and older age groups, with no clear trend observable based on visual inspection of the data (Figure 2).

**FIGURE 3:**
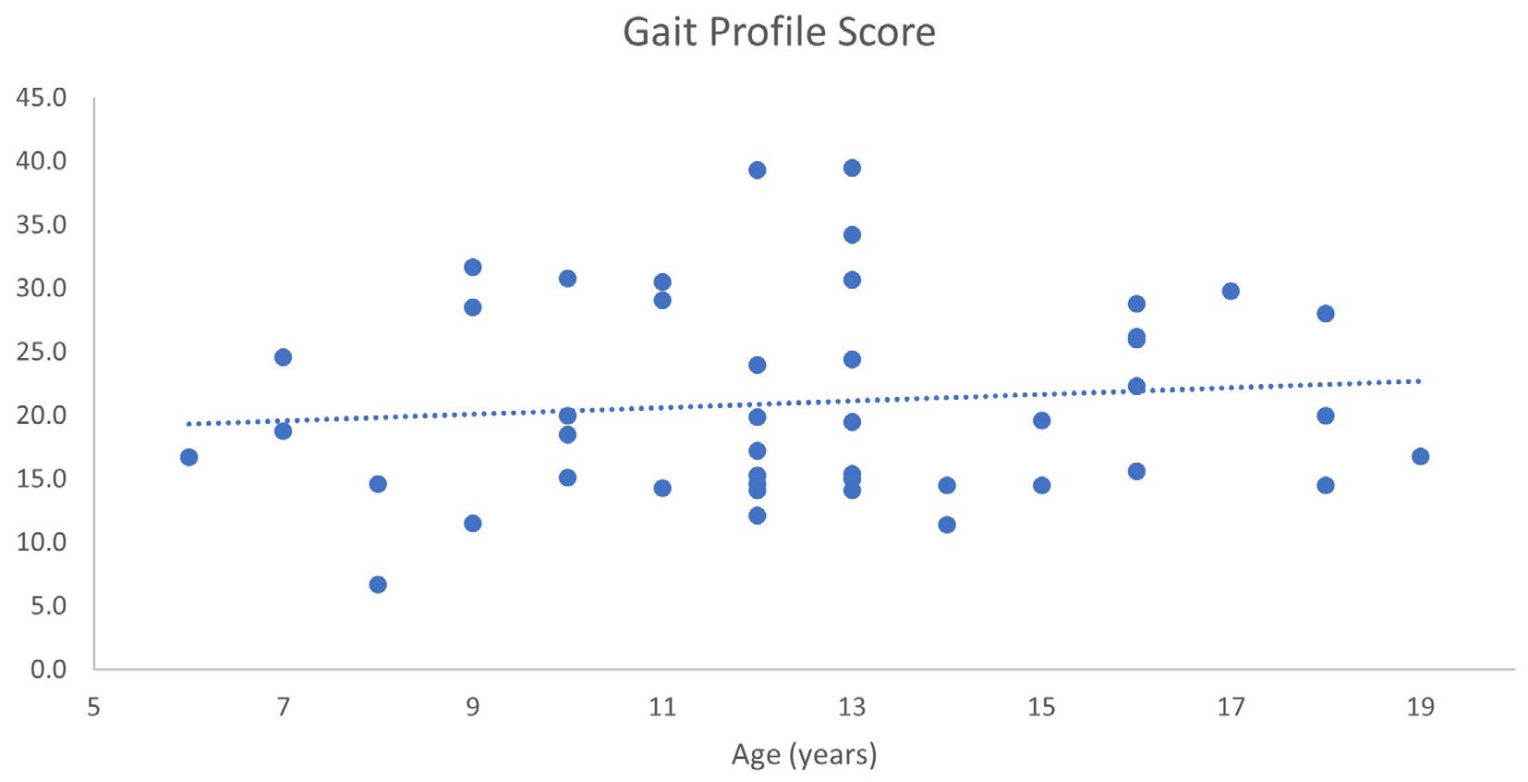
Gait Profile Score compared to age.

The GPS was highly variable, ranging from 6.7° to 39.5° between individuals, but showed no significant relationship with age, suggesting no overall change over time (*R* = 0.109, *p* = 0.469) (Figure 3).

**FIGURE 4:**
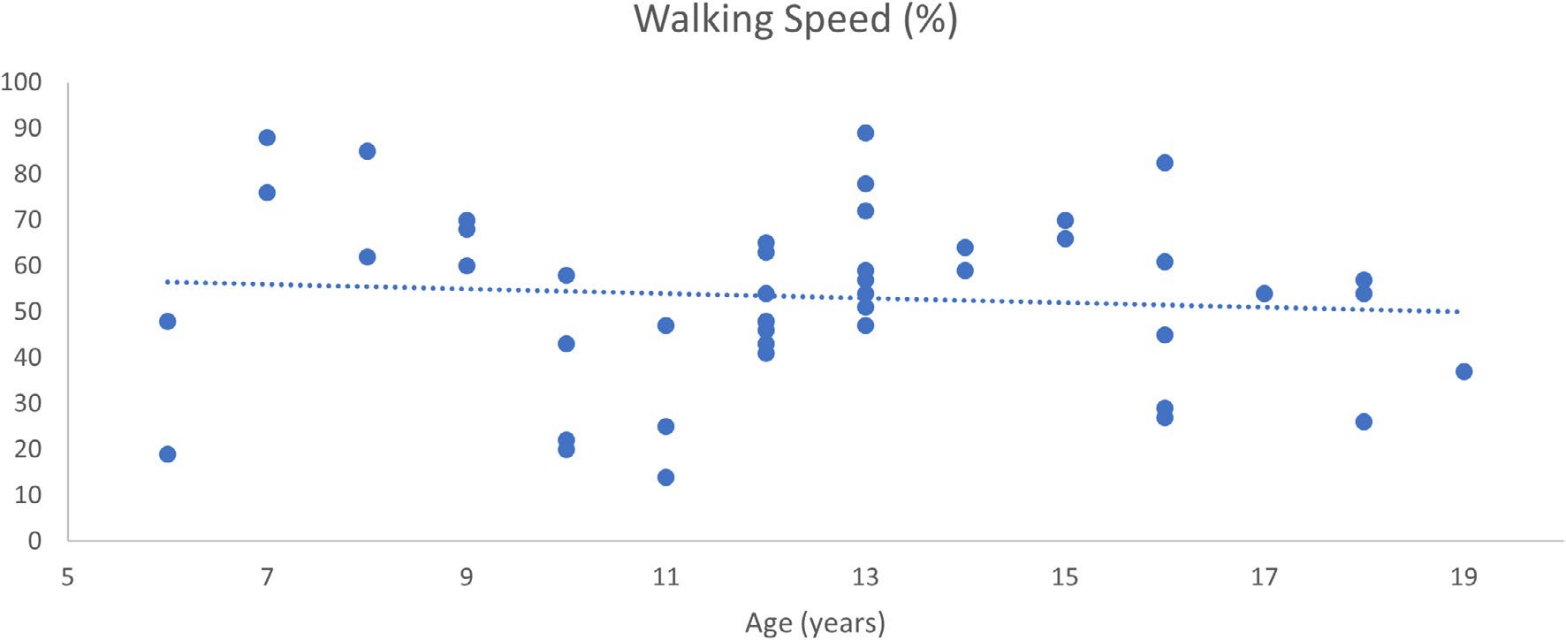
Walking speed normalized to leg length and reported as percentage of normal compared to age.

Walking speed was similarly variable, ranging from 14% to 89% of normal speed. There was again no significant relationship between speed and increasing age (*R* = −0.088, *p* = 0.562) (Figure 4).

**FIGURE 5:**
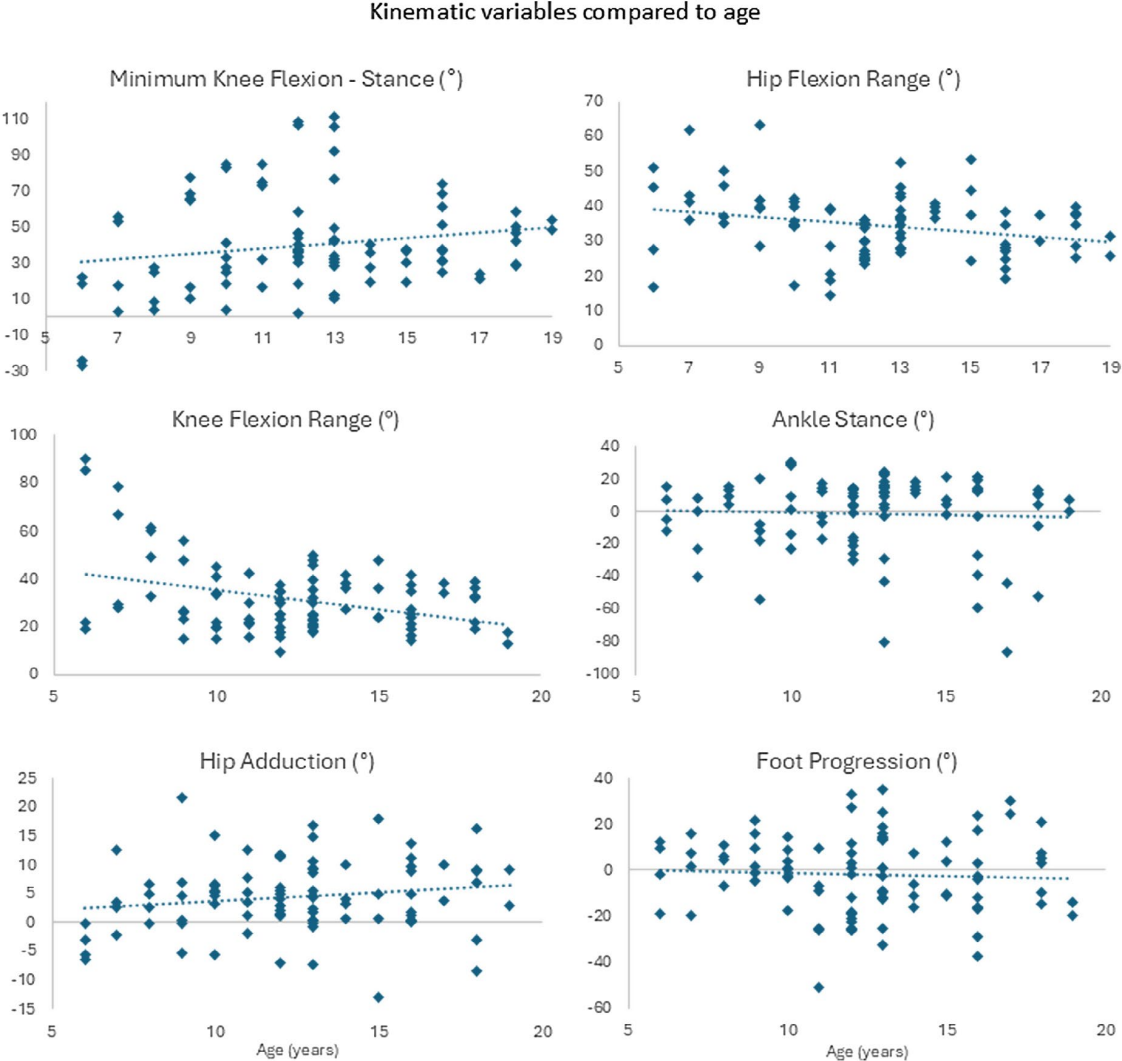
Specific gait variables compared to age.

The individual gait parameters assessed also showed large inter‐individual variation (hip flexion range: 14.5° to 63.5°; average hip adduction: 13.0° to 21.8°; maximum knee extension: −26.8° to 111.4°; knee flexion range: 9.6° to 90.1°; ankle dorsiflexion stance: −86.6° to 30.4°; foot progression: −50.5° to 35.4°) with only hip and knee flexion range showing a significant, negative relationship with age (maximum knee extension *R* = 0.175, *p* = 0.098; hip flexion range *R* = −0.263, *p* = 0.012; knee flexion range *R* = −0.356, *p* < 0.001, ankle dorsiflexion in stance *R* = −0.052, *p* = 0.626; hip adduction *R* = 0.159, *p* = 0.132, and foot progression angle *R* = −0.050, *p* = 0.636) (Figure 5).

## DISCUSSION

This is the first study to present a cross‐sectional assessment, including clinical examination and gait data, of ambulatory children with bilateral CP at different ages who have never previously presented to a health profession and have received no previous treatment. The data helps to understand what the true natural history of this condition is.

The results are highly heterogeneous, reflecting the large variability that is naturally present in this condition. There is general agreement in the literature over the typical evolution of ambulation in CP over time. The prevailing view is that children with bilateral CP tend to walk with equinus and near normal knee extension when they are young. Over time, plantarflexor contracture leads to ‘escape’ heel valgus and secondary planovalgus foot deformity and midfoot break. This, along with tightness in the knee flexors, weakness of the extensor mechanism of the knee, and rotational deviations, leads to progressive flexed knee (crouch) gait. However, the data from this study reveals large variation in physical exam measures, foot posture, and gait patterns in this population, with no clear trend with age, in the absence of any intervention. Perhaps the age‐related trends that have commonly been observed in the literature are associated more with treatment effects than natural evolution of the condition.

The results suggest that the clinical examination variables presented did not demonstrate clear association with age. There was a small but not statistically significant reduction in passive knee extension with age. It should be noted, however, that the two youngest participants displayed significant passive knee hyperextension which could have influenced the results. The incidence of different types of foot pathology also seemed to be independent of age. Of note, equinovarus feet were not more prevalent in the younger group, nor were planovalgus feet more prevalent in the older group, as would commonly be expected, again highlighting the natural heterogeneity of this population. Around half of the participants had significant asymmetry in their foot posture.

Walking patterns were shown to be highly variable between children, and overall gait pathology was not significantly associated with increasing age. However, some specific gait variables, including range of motion at the hip and knee in the sagittal plane, significantly reduced with age. Visual assessment of the data suggests that there is a plateau around 9 to 10 years of age. There is a tendency to increasing crouch (increased knee flexion during stance phase), which is consistent with the slightly reduced passive knee range seen in the physical examination; however, these findings were not statistically significant, likely because of the limited sample size. Of note, there were 6‐ and 7‐year‐olds who were already walking in significant crouch, and there were adolescents in their late teens with near normal knee extension, and an equinus‐type gait. Overall, the theory that children progress from equinus gait to crouch gait over time cannot be supported or refuted by these results.

The GPS has been widely reported in studies involving gait analysis for children with CP. It is interesting to compare the results from this study to those reported in the literature. Other available studies report on children from developed health care contexts, and therefore all children have likely received ‘typical’ neurological and orthopaedic management throughout their childhood. Four representative studies from the literature that report GPS are summarized in Table 1.^18^, ^19^, ^20^ These included children who had received ‘typical’ management throughout their childhood, mostly as a baseline before receiving multi‐level surgery. Untreated children with CP appear to demonstrate a similar pattern to those reported in the literature, with increasing severity of gait deviations associated with increasing GMFCS level. While there appear to be large differences in the mean GPS between those reported in the literature and the results of this untreated cohort, it is important to note that there is also a significant overlap in GPS scores between these cohorts. Some participants in this untreated cohort have a comparable GPS to those who have received treatment throughout their childhood, while some present with significant orthopaedic deformity, resulting in elevated GPS.

**TABLE 1:** GPS results from the literature compared to the results from this study.

Study	Number of participants	Mean age (years:months)	Male:female	GMFCS level	GPS (SD), °
Robinson et al.^18^				I	10.0 (2.8)
				II	11.8 (2.8)
				III	15.8 (4.8)
	151	13:4	NR	I–III	12.5 (NR)
Rutz et al.^19^	121	10:8	73:48	II–III	15.5 (3.8)
Dreher et al.^8^	19			I	13.4 (3.0)
	144			II	15.5 (4.3)
	68			III	19.0 (5.0)
	231	9:1	142:89	I–III	16.3 (4.8)
Baker et al.^20^	74			I	8.1 (2.4)^a^
	142			II	10.4 (4.2)^a^
	52			III	13.9 (4.3)^a^
	268		NR	I–III	10.8 (NR)
Data from this study	3	10:8	1:2	I	10.9 (3.9)
	32	12:5	23:9	II	20.4 (7.7)
	11	12:9	7:4	III	25.4 (5.7)
	46	12:5	31:15	I–III	21.0 (7.8)

Abbreviations: GMFCS, Gross Motor Function Classification System; GPS, Gait Profile Score; NR, not reported.

^a^ Interquartile range reported instead of standard deviation (SD).

In agreement with previous studies, it appears that the ranges of motion at the hip and knee during gait tend to reduce with age in this population that have not received any intervention. This links with the trend towards increasing severity of knee contractures with age, also demonstrated in this cohort. Therefore, it would be reasonable to prioritize interventions that maintain and/or improve range of hip and knee extension in this population. However, it should be noted that increasing crouch during walking can also be caused by multiple other factors including plantarflexor weakness; insufficiency in the quadriceps; torsional deformities in the long bones of the lower limbs; and/or weakness of the hip extensors. The cause of the crouch gait should therefore be thoroughly investigated before initiating treatment.

**FIGURE 6:**
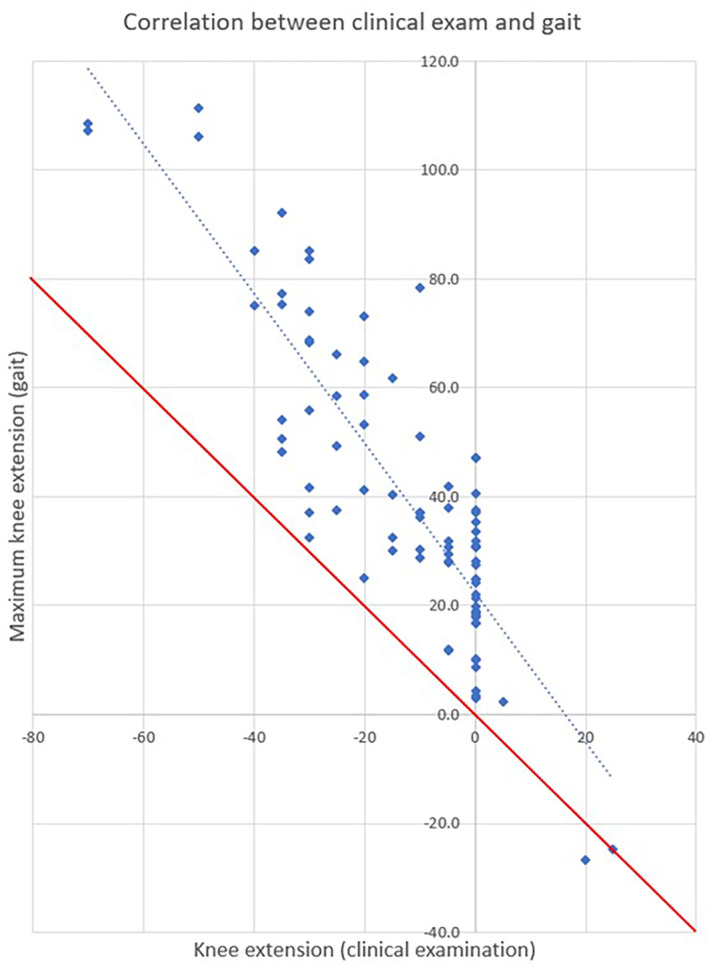
Correlation between maximum knee extension during gait (mid‐stance) and maximum knee extension achieved passively on clinical examination. The dashed line is the linear best fit of the regression. The solid line represents the 1‐to‐1 relationship in the data. Data points above this line indicate the participant walked with a greater degree of knee flexion during gait than was measured during the clinical examination.

In this population, 39 out of the 46 participants exhibited a crouch gait pattern, with the other participants showing a ‘jump’ pattern (knee flexion at initial contact with near normal knee extension in mid‐stance), a knee hyperextension pattern, or a very asymmetric pattern. Interestingly, on average the maximum knee extension during gait for each participant in this study was 27° less than the passive knee extension exhibited during the clinical examination. This suggests that the severity of crouch exhibited is at least in part dictated by factors other than passive knee extension (Figure 6).

In contrast with other studies,^4^, ^5^ there was no clear relationship established between hip adduction, ankle dorsiflexion, or foot progression angles during gait and increasing age. All these variables demonstrated large inter‐individual variability. Based on this data, it cannot be inferred that these variables deteriorate over time. Similarly, the physical examination variables of ankle dorsiflexion and femoral anteversion showed no clear relationship with age. Understanding which variables are likely to change with age, and which are more likely to remain relatively static, is useful in deciding between treatment options.

There are several limitations associated with this study. Similar to previous studies, ethical considerations prohibit controlled, longitudinal assessment in this cohort. Therefore, the study was necessarily cross‐sectional in nature. In addition, the data are potentially biased by only including children who were referred to the gait laboratory, which largely reflects those being considered for orthopaedic surgery. However, this limitation is common to all studies that include data collected in a gait laboratory, so the information is comparable to data from other gait laboratories around the world. A prospective study that includes a broader range of participants would help address this limitation. Given this is the only known data available on completely untreated children with CP, this still adds valuable information to the literature. The available data in the literature reporting on gait patterns in CP all originate from developed health care contexts since this is where gait laboratories are generally situated. This makes it challenging to compare to the data from this cohort, as there could be several factors that result in differences between settings. It is possible there are differences in the aetiology of CP between contexts. There are a few studies that suggest a lower rate of preterm births and higher incidence of birth asphyxia and postnatal infections^21^, ^22^ in sub‐Saharan African countries, which could result in a different presentation of CP. In addition, differences in environment, culture, schooling, and resource availability are likely to also affect motor development. A CP register that is specific to sub‐Saharan Africa could help develop this knowledge.^23^ Given these limitations, the focus of this study was on the association of gait patterns with age, and only observational comparisons were made between GPS results in the literature and those reported in this study. Clearly, these must be interpreted with caution.

The results of this study provide further evidence characterizing how bilateral CP is likely to develop with age. Our data provide baseline natural history information, against which the results of interventions can be compared. It is hoped that this will inform future research assessing outcomes. It will also guide decision‐making in the management of this condition.
